# Construction of polycistronic baculovirus surface display vectors to express the PCV2 Cap(d41) protein and analysis of its immunogenicity in mice and swine

**DOI:** 10.1186/s13567-020-00836-3

**Published:** 2020-09-09

**Authors:** Ya-Yi Chen, Wei-Chen Yang, Yu-Kang Chang, Chi-Young Wang, Wei-Ru Huang, Jyun-Yi Li, Kuo-Pin Chuang, Hung-Yi Wu, Ching-Dong Chang, Brent L. Nielsen, Hung-Jen Liu

**Affiliations:** 1 Department of Stomatology, Tung’s Taichung MetroHarbor Hospital, Taichung, Taiwan; 2 Medical Biotechnology, National Chung Hsing University, Taichung, 402 Taiwan; 3 Institute of Molecular Biology, National Chung Hsing University, Taichung, 402 Taiwan; 4 Department of Medical Research, Tung’s Taichung MetroHarbor Hospital, Taichung, Taiwan; 5 Department of Nursing, Jen-Teh Junior College of Medicine and Management, Hou-Long, Taiwan; 6 Department of Veterinary Medicine, National Chung Hsing University, Taichung, 402 Taiwan; 7 Graduate Institute of Animal Vaccine Technology, National Pingtung University of Science and Technology, Pingtung, 912 Taiwan; 8 Department of Veterinary Medicine, National Pingtung University of Science and Technology, Pingtung, 912 Taiwan; 9 Department of Microbiology and Molecular Biology, Brigham Young University, Provo, UT USA; 10 The iEGG and Animal Biotechnology Center, National Chung Hsing University, Taichung, 402 Taiwan; 11 Rong Hsing Research Center for Translational Medicine, National Chung Hsing University, Taichung, 402 Taiwan; 12 Translational Medicine, National Chung Hsing University, Taichung, 402 Taiwan; 13 Department of Life Sciences, National Chung Hsing University, Taichung, Taiwan

**Keywords:** PCV2, Cap protein, baculovirus surface display vectors, 4Cap(d41) vaccine, virus neutralization test, cellular immune response, CD4^+^ T cells, IFN-γ

## Abstract

To increase expression levels of the PCV2 Cap(d41) protein, novel baculovirus surface display vectors with multiple expression cassettes were constructed to create recombinant baculoviruses BacSC-Cap(d41), BacDD-2Cap(d41), BacDD-3Cap(d41), and BacDD-4Cap(d41). Our results reveal that the recombinant baculovirus BacDD-4Cap(d41) was able to express the highest levels of Cap(d41) protein. Optimum conditions for expressing the PCV2 Cap(d41) protein were determined, and our results show that 10^7^ of Sf-9 infected with the recombinant baculovirus BacDD-4Cap(d41) at an MOI of 5 for 3 days showed the highest level of protein expression. Mice immunized with the 4Cap(d41) vaccine which was prepared from the recombinant baculovirus-infected cells (10^7^) elicited higher ELISA titers compared to the Cap (d41) vaccine. The 4Cap(d41) vaccine could elicit anti-PCV2 neutralizing antibodies and IFN-γ in mice, as confirmed by virus neutralization test and IFN-γ ELISA. Moreover, the swine lymphocyte proliferative responses indicated that the 4Cap(d41) vaccine was able to induce a clear cellular immune response. Flow cytometry analysis showed that the percentage of CD4^+^ T cells and CD4^+^/CD8^+^ ratio was increased significantly in SPF pigs immunized with the 4Cap(d41) vaccine. Importantly, the 4Cap(d41) vaccine induced an IFN-γ response, further confirming that its effect is through cellular immunity in SPF pigs. An in vivo challenge study revealed that the 4Cap(d41) and the commercial vaccine groups significantly reduce the viral load of vaccinated pigs as compared with the CE negative control group. Taken together, we have successfully developed a 4Cap(d41) vaccine that may be a potential subunit vaccine for preventing the disease associated with PCV2 infections.

## Introduction

Porcine circoviruses (PCVs) are very small non‐enveloped animal viruses with circular single‐stranded DNA genomes of approximately 2 kb, which belong to the genus *Circovir*us in the family *Circoviridae* [1]. In pigs, four circovirus species (PCV1, PCV2, PCV3, and PCV4) within the genus Circovirus have been identified [1–3]. PCV1 is non-pathogenic and exists in contaminated pig kidney cell lines (porcine kidney 15, PK-15) and various pig tissues [1], wherase PCV2 is pathogenic and can cause pigs to develop postweaning multisystemic wasting syndrome (PMWS) [3]. The genome contains two main open reading frames (ORFs), ORF1 and ORF2. The PCV2 capsid protein (Cap) is the major structural viral proteins encoded by ORF2, which contains specific epitopes and induces specific neutralizing antibodies in the host [4–6]. Several reports suggested that this gene has great potential as a recombinant vaccine [5, 7]. Typical PMWS includes progressive weight loss, dyspnea, diarrhea, lower jaw, jaundice, inguinal lymphadenopathy, and systemic lymphocyte depletion [8, 9]. Severe cases can lead to death, causing economic losses for pig farmers in various countries in the world [10, 11]. The first case of PMWS was discovered in North America in 1991 [12, 13], and similar case reports were subsequently submitted in Spain, the United States, Denmark, Ireland, and other countries [14–17]. A suspected case of PMWS was discovered in Taiwan in 1995, and was first confirmed in 1997 to be PMWS with a mortality rate of about 5–20% [18]. At present, PCV2 infection in Taiwan is very common, and the mixed infection with other viruses or bacteria is also very serious. Therefore, the development of vaccines for comprehensive epidemic prevention is still the most effective way to deal with this problem.

The aim of this study is to construct novel baculovirus surface display vectors with multiple expression cassettes to enhance the expression levels of the PCV2 Cap protein and to develop a Cap subunit vaccine. We found that the recombinant baculovirus BacDD-4Cap(d41) was capable of expressing higher levels of Cap(d41) protein than other recombinant baculoviruses. Optimum conditions for expressing the PCV2 Cap(d41) protein were determined, and it was found that 3 days post infection with an MOI 5 or 10 yield the highest levels of protein expression. Mice immunized with the Cap(d41) vaccine which was prepared from the 10^7^ of Sf-9 cell lysates infected with the BacDD-4Cap(d41) recombinant baculovirus at an MOI of 10 for 3 days had higher ELISA titers than that of cell numbers of 10^5^ and 10^6^. Furthermore, ELISA and virus neutralization test showed that the 4Cap(d41) vaccine could elicit anti-PCV2 neutralizing antibodies and cellular immune response in mouse and specific pathogen-free (SPF) pigs. An in vivo challenge study showed the 4Cap(d41)-immunized pigs had significantly lower PCV2 viremia than that of the negative control group. The 4Cap(d41) vaccine has promise as a potential subunit vaccine for preventing the disease associated with PCV2 infections.

## Materials and methods

### Cells and viruses

The Spodoptera frugiperda (Sf-9) cell lines were grown as monolayers in TNM-FH medium (Sigma, St. Louis, USA) supplemented with 10% heat-inactivated fetal bovine serum (FBS; Gibco-BRL, Gaithersburg, USA), 100 U/mL of penicillin and 100 μg/mL of streptomycin. Recombinant viruses were propagated and titered in Sf-9 cells. The PCV2 virus strain was kindly provided by Professor Chiu, National Pingtung University of Science and Technology, Taiwan. A PK-15 cell line free of PCV1 contamination was maintained in DMEM supplemented with 10% FBS. The PCV2 strain used for the in vitro virus neutralization test was propagated in PK-15 cells.

### Construction of novel baculovirus surface display vectors carrying multiple expression cassettes

The detailed procedures to construct novel baculovirus surface display vectors with multiple expression cassettes was described previously [19, 20]. To construct the baculovirus surface display vector pBacSC [21], plasmid pBacCE was constructed using pFast-Bac DUAL [22]. Sequences coding gp64 SS, His6, multiple cloning sites (Xho I, Xba I, PstI, and EcoRI) localized between His6 and baculovirus gp64 CTD, and baculovirus gp64 TM were inserted into pBacCE and the resultant plasmid was named pBacSC. The baculovirus surface display vector BacDual Display (BacDD)-EGFP was constructed previously [20] and used to create pBacDD-2Cap (d41), pBacDD-3Cap (d41), and pBacDD-4Cap (d41) constructs. The 41 amino acids at the N-terminus of the PCV2 Cap is a nuclear localization signals (NLS) [23]. The first 41 amino acids at the N-terminus of the PCV2 Cap protein were deleted and the truncated Cap gene of PCV2 was constructed into the pBacSC vector. To construct the pBacSC-Cap(d41) plasmid, the PCV2 Cap gene with a deletion of the first 123 bp was amplified by PCR using prime pair PCV2-Cap(d41)-XhoI and PCV2-Cap(d41)-PstI (accession number AY885225; Table 1) and subcloned into the XhoI/PstI sites of the pBacSC plasmid.

**Table 1:** **Primer pairs used in this study**

Primer name	Sequence in 5′–3′ direction	Location (nt)	Accession number	Product name	Length (bp)
Cap(d41)-XhoI (Forward)	**XhoI** AGC***CTCGAG***ATGACGTATCCAAGG	124–702	AY885225	Cap(d41)	579
Cap(d41)-BsiWI (Reverse)	**PstI** TCAT***CTGCAG***TGAGGGTTTAA GTG				
Cap-(d41)-NotI (Forward)	**NotI** ***GCGGCCGC***TATGAATGGCATC TTCAACACC	124–702	AY885225	Cap(d41)	579
Cap(d41)-SalI (Reverse)	**SalI** ***GTCGAC***TATGGGTTTAAGTGG GGGGTC				
Cap(d41)-XhoI (Forward)	**XhoI** ***CTCGAG***ATGAATGGCATCTTC AACACC	124–702	AY885225	Cap(d41)	579
Cap(d41)-BsiWI (Reverse)	**BsiWI** ***CGTACG***TATGGGTTTAAGT GGGGGGTC				

To generate the BacDD-Cap(d41) plasmid, the Cap(d41)-gp64(TM-CTD) fragment from the pBacSC-Cap(d41) plasmid was excised by XhoI/KpnI digestion, and inserted into the XhoI/KpnI sites in the pBacDD-EGFP vector. To generate the pBacDD-2Cap(d41) plasmid, the PCV2 Cap gene with a deletion of the first 123 bp was amplified using prime pair PCV2-Cap(d41)-NotI and PCV2-Cap(d41)-SalI (Table 1) and subcloned into the NotI/Sa1I sites of the pBacDD-Cap(d41) vector. To create the pBacDD-4Cap(d41) plasmid, the Gem-T-easy-DD vector with two expression cassettes was constructed as described previously [20]. Two restriction enzyme sites MluI and BsiwI were included in this vector as described previously [20]. To construct the pGem-T-easy-DD-2Cap(d41) plasmid, pBacDD-2Cap(d41) was digested with KpnI and HindIIII to obtain the Cap(d41)-gp64(TM-CTD) fragment. The resultant fragment was then subcloned into the multiple cloning sites (KpnI and Hind III) of the pGem-T-easy-DD vector.

To construct the pBacDD-4Cap(d41) plasmid containing four expression cassettes, two expression cassettes containing the Cap(d41) gene from the pGem-T-easy-DD-2Cap(d41) plasmid were digested by MluI and BsiWI, and the generated fragment was inserted into the corresponding sites of the pBacDD-2Cap(d41) plasmid. To construct the pBacDD-3Cap(d41) plasmid, pBacDD-4Cap(d41) plasmid was digested with BstZ17I to remove one expression cassette. The completed vector was named pBacDD-3Cap (d41).

### Preparation of recombinant bacmid DNA and construction of recombinant baculoviruses

**Figure 1:**
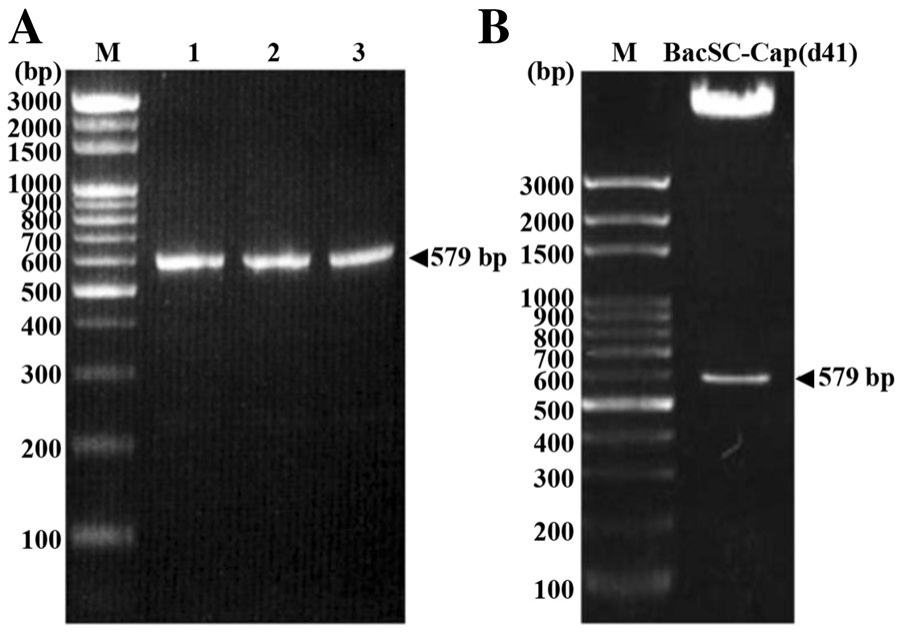
**DNA electrophoresis analysis of the PCV2-Cap (d41) gene PCR product and confirmation of the pBacSC-Cap (d41) plasmid. A** Lane M represents DNA marker (Bio-100 bp DNA ladder); lanes 1-3 represent PCR amplified PCV2-Cap (d41) gene fragments, which have restriction enzyme cleavage positions (Lane 1: XhoI/PstI, lane 2: NotI/SalI and lane 3: XhoI/BsiWI). The expected fragment size is 579 bp in length. **B** The pBacSC-Cap (d41) vector was cleaved by restriction enzymes XhoI and PstI to confirm the successful gene recombination, which was in line with the expected fragment size of about 579 bp in length.

The resultant constructs pBacSC-Cap(d41), pBacDD-2Cap(d41), pGem-T-easy-DD-2Cap(d41), pBacDD-3Cap(d41), and pBacDD-4Cap(d41) were verified by enzyme digestion and DNA sequencing (Figures 1B, 3A). Competent DH10Bac *E. coli* were then transformed with these recombinant plasmids and the non-recombinant plasmid pBacCE, respectively. The non-recombinant pBacCE was used as a negative control. After two rounds of blue/white selection, recombinant bacmids were isolated from white colonies according to the manufacturer’s instructions (Invitrogen, Carlsbad, USA). The recombinant clones were then examined for the presence of the insert by PCR using Cap(d41) specific primers (Table 1). Positive colonies were cultured in order to isolate the bacmid DNA. Sf-9 cells were cultured at 27 °C in Sf-900 II SFM. 9 × 10^5^ cells were seeded in 35-mm wells of a six-well plate and allowed to attach for 1 h before transfection. Transfected Sf-9 cells were incubated for 5 h at 27 °C and replaced with fresh medium. After incubation for 48 h at 27 °C, recombinant viruses were selected based on GFP expression and purified by three rounds of plaque isolations. Individual recombinant viruses were titered by plaque assay and high titer stocks were utilized for infecting the cells.

### Sodium dodecyl sulfate polyacrylamide gel electrophoresis (SDS-PAGE) and western blot assays

Infected cell lysates were subjected to 12% SDS-PAGE and transferred to nitrocellulose membranes. Two primary antibodies were used to detect the Cap(d41) protein by western blot. Expressed proteins were probed by anti-PCV2 sera for the Cap(d41) protein. The secondary antibody for the reaction was anti-mouse IgG conjugated to HRP (1:3000 dilution, Invitrogen). Protein bands were visualized using the ECL chemiluminescence kit (Amersham Pharmacia Biotech, New Territories, Hong Kong). Infected cell lysates were subjected to 10% SDS-PAGE and transferred to nitrocellulose membranes. Two primary antibodies (anti-His6 monoclonal antibody 1:3000 dilution and anti-CPV-2 polyclonal antibody 1:1000 dilution) were used to detect CPV VP2 protein by western blot. The secondary antibody was goat anti- mouse IgG conjugated to HRP (1:5000 dilution; KPL, Gaithersburg, Maryland, USA). The protein bands were visualized by the ECL chemiluminescence system on Hyper-Max films, as recommended by the manufacturer (Amersham Pharmacia Biotech, Hong Kong).

### Comparison of the expression levels of the PCV2 Cap(d41) protein by different recombinant baculoviruses and optimum conditions for production of the PCV2 Cap(d41) protein

To compare the expression levels of Cap(d41) expressed by different genetic recombinant baculovirus, 10^7^ Sf-9 cells were cultured in a 24-well plate, and then individually infected with recombinant baculoviruses BacSC-Cap(d41), BacDD-2Cap(d41), BacDD-3Cap(d41), and BacDD-4Cap(d41) at an MOI of 5, respectively. After 3 days of infection, cell lysates were collected and the expression levels of the PCV2 4Cap(d41)protein were analyzed by western blot assays.

To increase expression levels of the Cap (d41) protein, optimum conditions for recombinant baculovirus transfection were examined. In this work, various MOI (1, 5, and 10 MOI), infection times (2–4 days), and different cell numbers (10^5^, 10^6^, and 10^7^) for culture of recombinant baculoviruses BacSC-Cap(d41), BacDD-2Cap (d41), pBacDD-3Cap (d41), and pBacDD-4Cap (d41) were tested. As mentioned above, Sf-9 cells were grown at 27 °C in Grace’s insect media (Invitrogen) supplemented with 10% heat inactivated FBS (Gibco-BRL), 100 μg/mL of penicillin and 100 μg/mL of streptomycin. After 3 days of infection, cells were collected and analyzed by western blot assays. To further examine the efficacy of the immune response with different immunogens, different numbers (10^5^, 10^6^ and 10^7^) of Sf-9 cells were infected with the recombinant baculoviruses BacDD-4Cap (d41) and BacSC-Cap (d41) at an MOI of 10 for 3 days, respectively. The respective cell lysates were used to prepare Cap(d41) and 4Cap(d41) vaccines to immune mice. The immune experiments were divided into 6 groups (n = 5 for each group). These groups included the negative control group (PBS and CE), positive control group (commercial porcine circovirus vaccine, Ingelvac^®^ Circo FLEXTM), and experimental groups (BacDD-Cap (d41) and BacDD-4Cap (d41) vaccines). Mice were immunized by the above vaccines via intraperitoneal injection. The same volume of complete adjuvant (Freund’s adjuvant complete adjuvant) and the respective cell lysates (BacCE, BacDD-Cap(d41), and BacDD-4Cap(d41) were mixed in the first immunization and incomplete adjuvant was used in the second immunization. Two weeks after the first immunization, the second dose was given. The days post immunization (days post vaccination; dpv) start from the first immunization, and blood samples were taken by tail blood collection in the fourth week after immunization and at 2 weeks after the booster. Blood samples were centrifuged at 3000 rpm for 15 min at 4 °C to separate the serum, and the supernatant was stored at − 20 °C for subsequent experiments. Antibody titers were measured by enzyme-linked immunosorbent assay (ELISA).

To establish an ELISA for detection of the PCV2 Cap(d41) antibody, the optimal dilutions of antigen and serum were determined by checker board titration with PCV2-positive and -negative swine sera [24]. For expression of Cap(d41) in *E. coli*, the PCV2 Cap gene with a deletion of the first 123 bp was amplified by PCR using prime pair PCV2-Cap(d41)-XhoI and PCV2-Cap(d41)-PstI (Table 1) and subcloned into the expression vector pET32a. The procedures for expression of Cap(d41) in *E. coli* have been described previously [25, 26]. Briefly, the recombinant plasmid was transformed into *E. coli* BL21(DE3). The transformed *E. coli* cells were grown in Luria–Bertani (LB) broth with 100 μg/mL of ampicillin at 37 °C to an optical density of 0.6 and then induced with 0.4 mM of IPTG for 5 h at 28 °C. Cells were harvested by centrifugation, followed by resuspension in pET system lysis buffer (20 mM Tris–HCl pH 8, 300 mM NaCl, 0.2 mM PMSF, 10% glycerol, 5 mM imidazole) and sonicated. Cell suspensions were centrifuged at 12,000 × *g* for 20 min at 4 °C. Each supernatant was applied to a nickel column. After washing beads with 150 mL washing buffer, TrxA-His-tagged Cap(d41) fusion proteins were eluted from the affinity column with elution buffer (20 mM Tris–HCl pH 8, 300 mM NaCl, 0.2 mM PMSF, 10% glycerol, 200 mM imidazole). Finally, purified fusion proteins were changed to PBS buffer with Amicon Ultra 0.5-mL 10 k filters (Millipore). Samples were stored at − 80 °C for further ELISA experiments. After adding the purified Cap(d41) protein to the coating buffer, 100 μL was added into each well and incubated at 4 °C for 16 h. Each well of the plates was coated with the Cap(d41) protein (50 ng) in 100 μL of coating buffer at 4 °C for 18 h. After that, the coating buffer was removed, 100 μL of blocking buffer (5% skim milk) was added to each well and incubated at room temperature for 1 h. After washing three times with PBS (containing 0.1% Tween 20), 100 μL of serum samples were diluted and added into each well and incubated for 1 h at 37 °C. The plates were washed with PBS three times. For detection of the bound antibodies, 100 μL of goat-anti-mouse IgG-HRP antibodies (KPL) were added and the plates were shaken at room temperature for 1 h. Goat-anti-mouse IgG-horseradish peroxidase G-A-M IgG-HRP conjugate was used at a dilution of 1:5000. After washing with PBS 3 times, 100 μL of ABTS solution was added to each well for 15 min in the dark and the reaction was stopped by adding 100 μL of stop solution (20% SDS) to each well to stop the color reaction. The OD_405_ nm value was measured with an ELISA reader.

### Immunization of SPF pigs and measurement of CPV Cap(d41) titers

2-month-old SPF pigs were immunized at the base of the ear by the intramuscular route with CE, 4Cap(d41), and commercial vaccines, respectively. The pig serum was collected every week after the first immunization. In the eighth week after the first immunization, all vaccinated pigs were intranasally injected with 2 mL of PCV2 (10^5.7^TCID_50_/mL). Sera samples were collected at 14 and 28 days after primary immunization for virus neutralization tests. Peripheral blood mononuclear cells (PBMCs) were isolated from the whole blood of immunized swine for lymphocyte proliferation assay at 42 days after primary immunization. The sera were used for examination of the ELISA titer. The sera were tested for the presence of Cap antibody titers using PCV2 antibody detection ELISA kit. All swine was confirmed without antibodies against PCV2 before used.

### Virus neutralization test

To prepare the vaccines, 5 × 10^7^ Sf-9 cells were infected with the recombinant baculoviruses BacSC-4Cap(d41) and BacCE at an MOI of 10 for 3 days, respectively, and the cell lysates were collected to prepare the 4Cap(d41) and CE vaccines. To examine neutralizing antibody titers in vaccinated pigs and mice, the immune experiments were divided into three groups (n = 3 for each group). These groups include the negative control group CE, commercial porcine circovirus vaccine (Ingelvac^®^ Circo FLEXTM), and 4Cap (d41) vaccine. The mice were immunized with the respective vaccines by intraperitoneal injection. The pigs were immunized with the respective vaccines formulated with ISA201by intramuscular injection. Two weeks after the first immunization, the second dose was given. PCV2 neutralizing titers of vaccinated pigs and mice were determined at 14 and 28 days or 35 days after primary vaccination. PCV2 neutralizing titers of vaccinated mice were detected at 35 days after primary vaccination.

To carry out the virus neutralization test, PK-15 cell lines (10^4^ cells/well) were cultured in 96-well plates. Briefly, sera (50 μL) from mice 35 days after vaccination were diluted in in twofold increments in DMEM medium (pH 7.0), starting with a dilution factor of 1:2. An equal volume of PCV2 (200 TCID_50_) was added to the serum samples and incubated at 37 °C for 1 h. The mixed solution (serum and PCV2 virus) was then inoculated into a 96-well plate containing 40–50% confluent PK-15 cells for 72 h. After washing three times by PBS, 90% acetone was added to each well at − 20 °C for 30 min, and the acetone was removed and wells were washed with PBS for 3 times. The culture plate was fixed with 90% acetone, dried and incubated with porcine anti-PCV2 polyclonal antibodies, followed by staining with the FITC-conjugated goat anti-porcine IgG (Invitrogen). Titers were determined as the reciprocal of the last serum dilution with 70% or greater fluorescent focus reduction in the infected cells under a fluorescent microscope. Finally, the neutralizing antibody titers were calculated by the Reed-Muench method [27].

### Lymphocyte proliferation assay

The lymphocyte proliferation assay was performed using peripheral blood mononuclear cells (PBMCs) of swine. PBMCs were isolated from the whole blood of immunized swine with a lymphocyte separation medium. After washing three times with PBS, the PBMCs were resuspended at 10^6^ cells/mL in RPMI-1640 supplemented with 10% FBS, and seeded in 96-well flat-bottom plates at 100 μL per well. Experimental groups were divided into commercial vaccine (10 μL), 4Cap(d41), and the negative control CE. These vaccines were added into the respective well and placed in a 37 °C incubator for 68 h followed by addition of 10 μL of MTT (3-(4,5-cimethylthiazol-2-yl)-2,5-diphenyl tetrazolium bromide; 5 mg/mL per well) in a 37 °C incubator for 4 h. Finally, the reaction was terminated by adding 100 μL of DMSO. The optical density (OD) was determined at 490 nm, and the stimulation index (SI) was calculated as follows: SI = mean OD of PCV2 stimulated cells/mean OD of unstimulated cells.

### Detection of CD4^+^ and CD8^+^ analysis of immunized pig PBMC cells by flow cytometry

At the sixth week after the first immunization, sera was collected to isolate PBMCs of each group of immunized pigs. After washing three times with PBS buffer, 10^6^ cells of PBMC were divided into 1.5 cc centrifuge tubes. FITC mouse anti-pig CD4a monoclonal antibody and PE mouse anti-pig CD8a monoclonal antibody (BD Biosciences, San Jose, USA) were added. The reaction was carried out in the dark at 4 °C for 30 min. After washing once, the cells were resuspended in PBS and analyzed by flow cytometry (BD FACSCalibur, USA). The percentages of CD4 ^+^ and CD8 ^+^ and the CD4^+^/CD8^+^ ratio of PBMC cells were analyzed.

### Analysis of IFN-γ and IL-4 by ELISA

Analysis of IFN-γ and IL-4 by ELISA was performed using the IFN-γ and IL-4 ELISA Kit (Uscn, USA), and murine IFN-γ ELISA KIT (Gen-Probe, San Diego, USA). Firstly, 100 μL of pig serum or mouse serum were added to the ELISA plates with pre-coated monoclonal antibody at 37 °C for 2 h. After washing with PBS three times, the plates were blocked with complete RPMI medium containing 10% fetal bovine serum (FBS) for 2 h at 37 °C. Finally, 100 μL of streptavidin-HRP was added to each well for 20 min at room temperature. After washing 3 times, 100 μL of 3,3′,5′,5′-tetramethylbenzidine (TMB) coloring agent was added to each well. Finally, the color development was initiated by adding 100 μLl of TMB buffer (100 μL/well) and terminated by adding 100 μL of H_2_SO_4_. The OD_405_ nm value was measured with an ELISA reader.

### Swine challenge test and real-time quantitative PCR

On the eighth week after the first immunization, all vaccinated pigs were intranasally injected with 2 mL of PCV2 (10^5.7^TCID_50_/mL). DNA was extracted from serum samples (containing PCV2 DNA) and analyzed by real-time quantitative PCR [28]. The sequences of primers are as follow: P5: 5′-GCTGAACTTTTGAAAGTGAGCGGG-3′, P6: 5′-TCAC ACAGTCTCAGTAGATCATCCCA-3′. The expected size of PCR product is 220 bp in length. The PCR amplified products were ligated into the pGEM-T easy vector, and then transformed into *E. coli* DH5α. Colonies were screened with Amp^+^, the DNA was extracted and amplified, the plasmid DNA concentration was measured, and the plasmid DNA was diluted tenfold into six gradients. As a standard, real-time fluorescent quantitative PCR was used to prepare a standard curve. Finally, the DNA samples extracted every week were analyzed by real-time quantitative PCR. The reaction volume was 20 μL, consisting of 10 μL of 2X QuantiFast SYBR Green PCR Master Mix, 2 μL of P5 primer (10 μM/μL), 2 μL of P6 primer (10 μM/μL), 2 μL of DNA template and 5  μL of RNase-free water. The reaction conditions were 95 °C for 5 min, 95 °C for 10 s, 60 °C for 1 min, for a total of 30 cycles, and an analysis curve was prepared after the reaction.

### Statistical analysis

All data were analyzed using independent sample *t* test and are expressed as averages of three independent experiments. A P value less than 0.05 was considered significant.

## Results

### Amplification of the PCV2 Cap(d41) gene and construction of pBacSC-Cap (d41) plasmid

**Figure 2:**
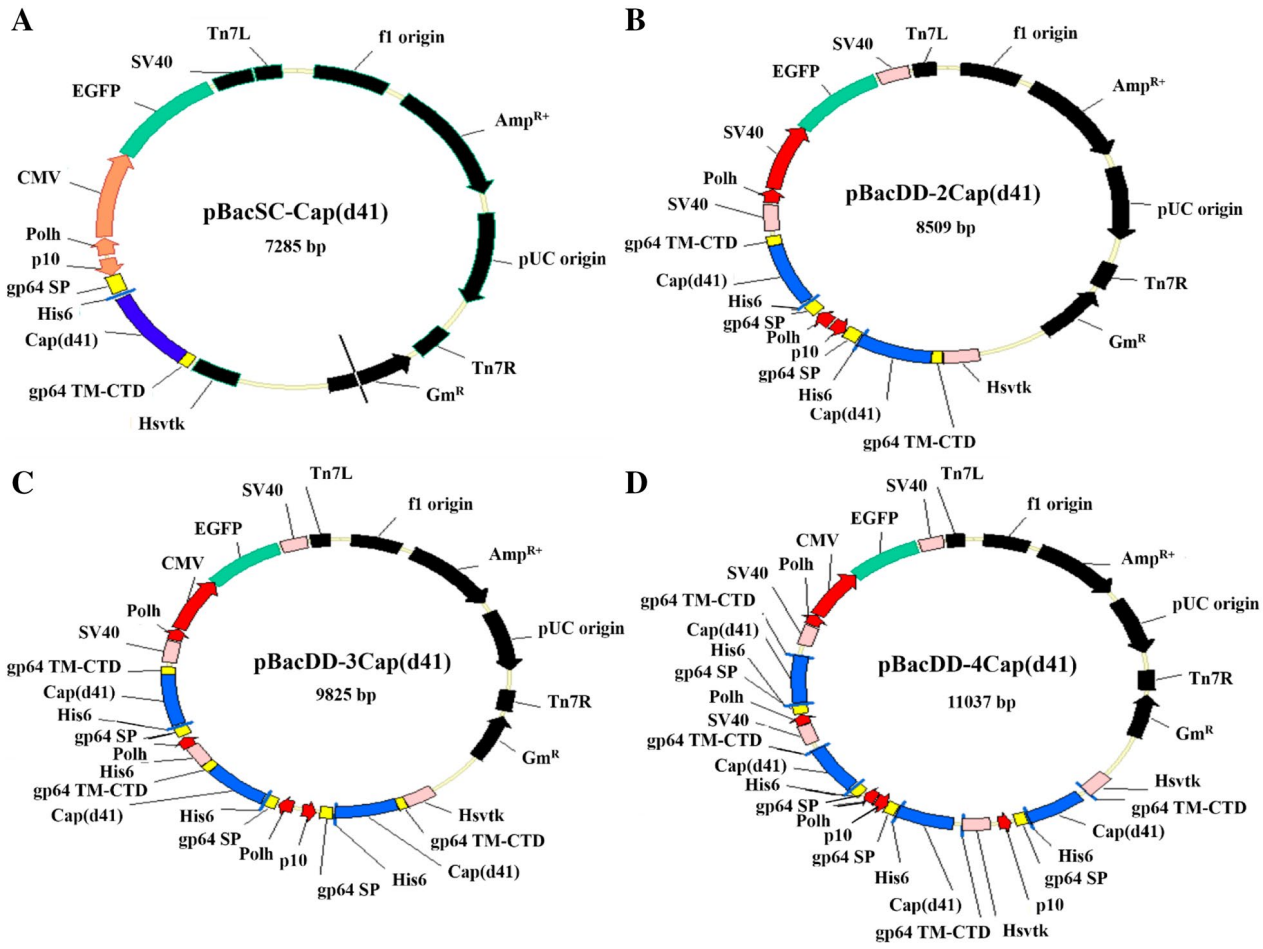
**Schematic illustration of novel baculovirus surface display vectors carrying multiple expression cassettes.** To increase the expression levels of the PCV2 Cap(d41) protein, four novel baculoviral vectors pBacSC, pBacDD-2, pBacDD-3, and pBacDD-4, which carry multiple expression cassettes were constructed. The Cap(d41)-gp64-SP-TM-CTD fragment was inserted into the respective baculoviral vectors and the resultant recombinant plasmids were named pBacSC-Cap(d41), pBacDD-2Cap(d41), pBacDD-3Cap(d41), and pBacDD-4Cap(d41) (**A**–**D**).

In this study, the PCV2 Cap gene with deletion of the first 123 bp was amplified by PCR. The expected size of the PCR products is 579 bp in length (Figure 1A). The resulting gene is named Cap(d41). The PCR product was subcloned into the restriction sites of *XhoI* and *PstI* in the baculovirus surface display vector (pBacSC) [21] and the resultant plasmid was named as pBacSC-Cap(d41) (Figure 1). To further confirm the recombinant pBacSC-Cap(d41) plasmid contained the PCV2 Cap(d41) gene, this vector was digested with restriction enzymes *XhoI* and *PstI*. As shown in Figure 1B, the expected fragments are seen. The pBacSC-Cap(d41) plasmid was also sequenced to confirm their authenticity. To improve the recombinant virus selection, enhanced green fluorescent protein (EGFP) coding sequences were subcloned into the corresponding site in plasmid BacSC under the strong viral polyhedron (polh) promoter (Figure 2A).

### Construction of novel baculovirus surface display vectors carrying multiple expression cassettes for high level expression of the PCV2 Cap(d41) protein

**Figure 3:**
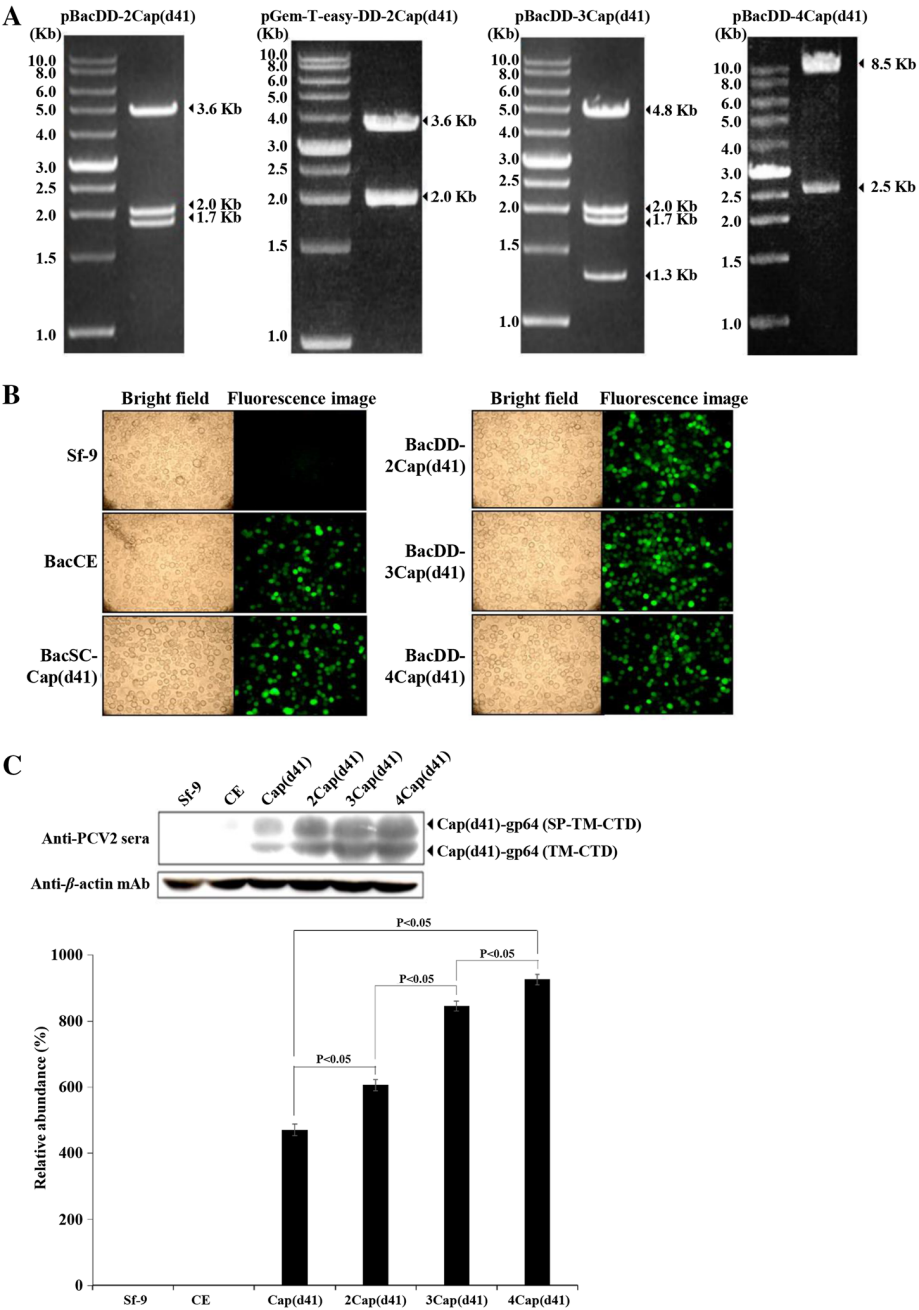
**Confirmation of recombinant baculovirus surface vectors and expression levels of the Cap (d41) protein using the respective baculovirus surface vectors. A** Four recombinant plasmids pBacDD-2Cap(d41), pGem-T-easy-DD-2Cap(d41), pBacDD-3Cap(d41), and pBacDD-4Cap(d41) were digested with the respective restriction enzymes for confirming the presence of the Cap(d41) gene of PCV2. **B** Images of BacSC-Cap(d41), BacDD-2Cap(d41), BacDD-3Cap(d41), and BacDD-4Cap(d41) recombinant baculovirus-infected Sf-9 cells at 3 days post-infection. All images were magnified at 200×. **C** Confirmation of the expression of Cap(d41)-gp64-TM-CTD protein in Sf-9 cells. The cells were infected with recombinant viruses BacCE, BacSC-Cap(d41), BacDD-2Cap(d41), BacDD-3Cap(d41), and BacDD-4Cap(d41), respectively, at an MOI of 10, harvested 3 days post infection, and subjected to western blot assay using anti-PCV2 sera (lanes 2–6). The Cap(d41) protein has two different fragments (Cap(d41)-gp64-SP-TM-CTD with a molecular weight of 33 kDa; Cap(d41)-gp64-TM-CTD with a molecular weight of 28 kDa. Sf-9 cells and BacCE were used as the negative control. Signals in all western blots were quantified using Image J software. The levels in the Cap(d41) were considered one-fold. β-actin was used as an internal control for normalization. The expression folds indicated below each lane were normalized against values for Cap(d41). Data represent the mean ± SD. A p value less than 0.05 was considered significant.

In this study, baculovirus surface display vectors pBacDD-EGFP and pGem-T-easy-DD were constructed as described previously [20]. These vectors were used to create pBacDD-2Cap(d41), pBacDD-3Cap(d41), and pBacDD-4Cap(d41) constructs (Figure 2B–D). Three recombinant baculoviruses BacDual Display (BacDD)-2Cap(d41), BacDD-3Cap(d41), and BacDD-4Cap(d41) were further created to express His6-tagged Cap(d41) (with C-terminal gp64 TM-CTD). The resultant pBacSC-Cap (d41), pBacDD-2Cap(d41), pBacDD-3Cap(d41), and pBacDD-4Cap(d41) plasmids were used to generate BacSC-Cap (d41), BacDD-2Cap(d41), BacDD-3Cap(d41), and BacDD-4Cap(d41) recombinant baculoviruses using the Bac-to-Bac system (Invitrogen). To further confirm that these recombinant pBacDD-2Cap(d41), pBacDD-3Cap(d41), and pBacDD-4Cap(d41) plasmids contained the PCV2 cap(d41) gene, these vectors were digested with the respective restriction enzymes. As shown in Figure 3A, the expected fragments are seen. These vectors were also further sequenced to confirm their authenticity. Fluorescence images of Sf-9 cells infected with BacSC-Cap (d41), BacDD-2Cap(d41), BacDD-3Cap(d41), and BacDD-4Cap(d41) recombinant baculoviruses at 3 days post-infection are shown in Figure 3B. By comparing the expression levels of the PCV2 Cap(d41) proteins by different recombinant baculovirus, we found that the recombinant baculovirus BacDD-4Cap(d41) was able to express higher levels of the Cap(d41) protein than other recombinant baculoviruses (Figure 3C). Our results reveal that the protein expressed by the recombinant baculovirus display system can not only present on the cell membrane but also exist in the cytoplasm.

### Optimum conditions for production of the PCV2 Cap(d41) protein

**Figure 4:**
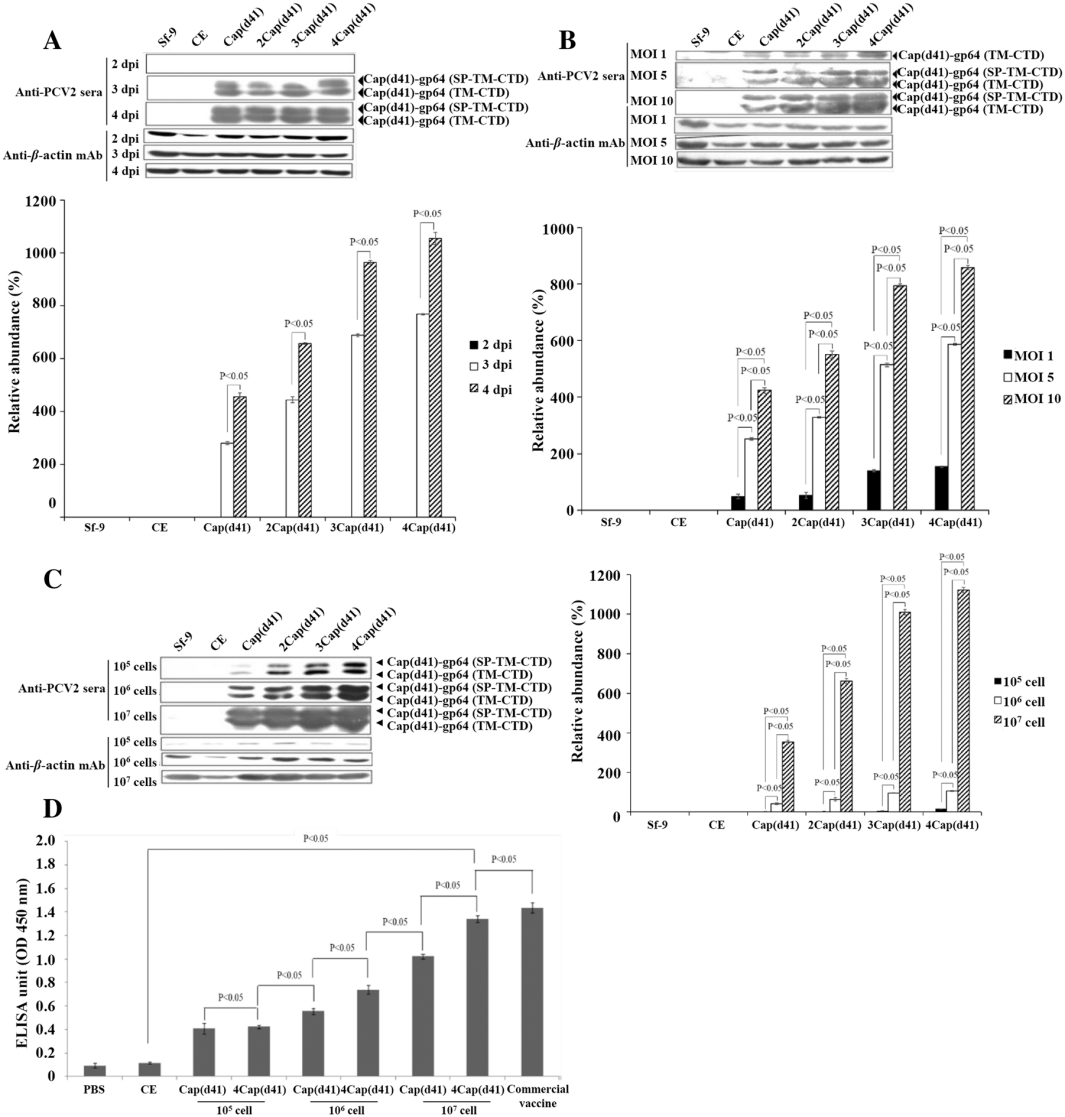
**Optimum conditions for production of the PCV2 Cap(d41) protein.** Effects of different infection times, MOI, and cell numbers for expressing the Cap(d41) protein were tested. **A**, **B** Cell numbers (10^7^) of Sf-9 cells were infected with MOI of 10 (**A**) or infected with different MOI (**B**) for 4 days. Cell numbers (10^7^) of Sf-9 cells were infected with different recombinant baculoviruses as indicated. The Sf-9 cell alone and CE recombinant baculoviruses were used as the negative control. Signals in all western blots were quantified using Image J software. β-actin was used as an internal control for normalization. Relative abundance (%) are shown. The results were calculated from the data shown in the upper portion of each panel. All data shown represent the mean ± SD calculated from three independent experiments. A student t-test was conducted for analysis and a value of P < 0.05 was considered statistically significant. **C** Expression levels of the PCV2 Cap(d41) protein in different cell numbers infected with recombinant baculovirus at an MOI of 10 for 4 days was examined. Signals in all western blots were quantified using Image J software. β-actin was used as an internal control for normalization. Relative abundance (%) are shown. The results were calculated from the data shown in the left portion of each panel. All data shown represent the mean ± SD calculated from three independent experiments. A student t-test was conducted for analysis and a value of P < 0.05 was considered statistically significant. **D** The immune effect of different doses of Cap(d41) protein and evaluation of the effective dose. Different cell numbers (10^5^, 10^6^ and 10^7^) of Sf-9 cells were infected with recombinant baculoviruses BacSC-Cap(d41) and BacDD-4Cap(d41), respectively, at an MOI of 10 for 3 days, respectively and cell lysates were collected to immunize mice. After two vaccinations, antibody titers were analyzed by ELISA. Data represent the mean ± SD. A p value less than 0.05 was considered significant.

To increase the expression/display levels of the PCV2 Cap(d41) protein, the optimum conditions for recombinant baculovirus transfection and cell growth were tested. As shown in Figure 4A, the conditions of an MOI of 10 and 4 day post infection have higher levels of PCV2 Cap(d41) protein expression while after 2 days of infection with insect cells, the PCV2 4Cap(d41) protein was still not detected. Among the MOI tested, both 5 and 10 MOIs yield higher levels of protein expression and are the optimal conditions for protein expression (Figure 4B). Furthermore, production of the PCV2 Cap(d41) protein in different cell numbers infected with recombinant baculovirus at an MOI of 10 was also examined. Our results reveal that higher levels of the PCV2 Cap(d41) protein were seen in both BacDD-4Cap(d41) and 10^7^ cell numbers (Figure 4C). Furthermore, we examined the immune effect of different doses of Cap(d41) protein and evaluated the effective dose. Different cell numbers (10^5^, 10^6^ and 10^7^) of Sf-9 cells were infected with recombinant baculoviruses at an MOI of 10 for 3 days, respectively and the cell lysates were collected to immunize mice. After two vaccinations, the antibody was collected and analyzed by ELISA. The results confirmed that commercial vaccine and the PCV2 Cap(d41) protein expressed by the BacDD-4Cap(d41) recombinant baculovirus can effectively induce higher ELISA titers in mice (Figure 4D).

### 4Cap(d41) and commercial vaccines induced neutralizing antibody in mice and SPF pigs

**Figure 5:**
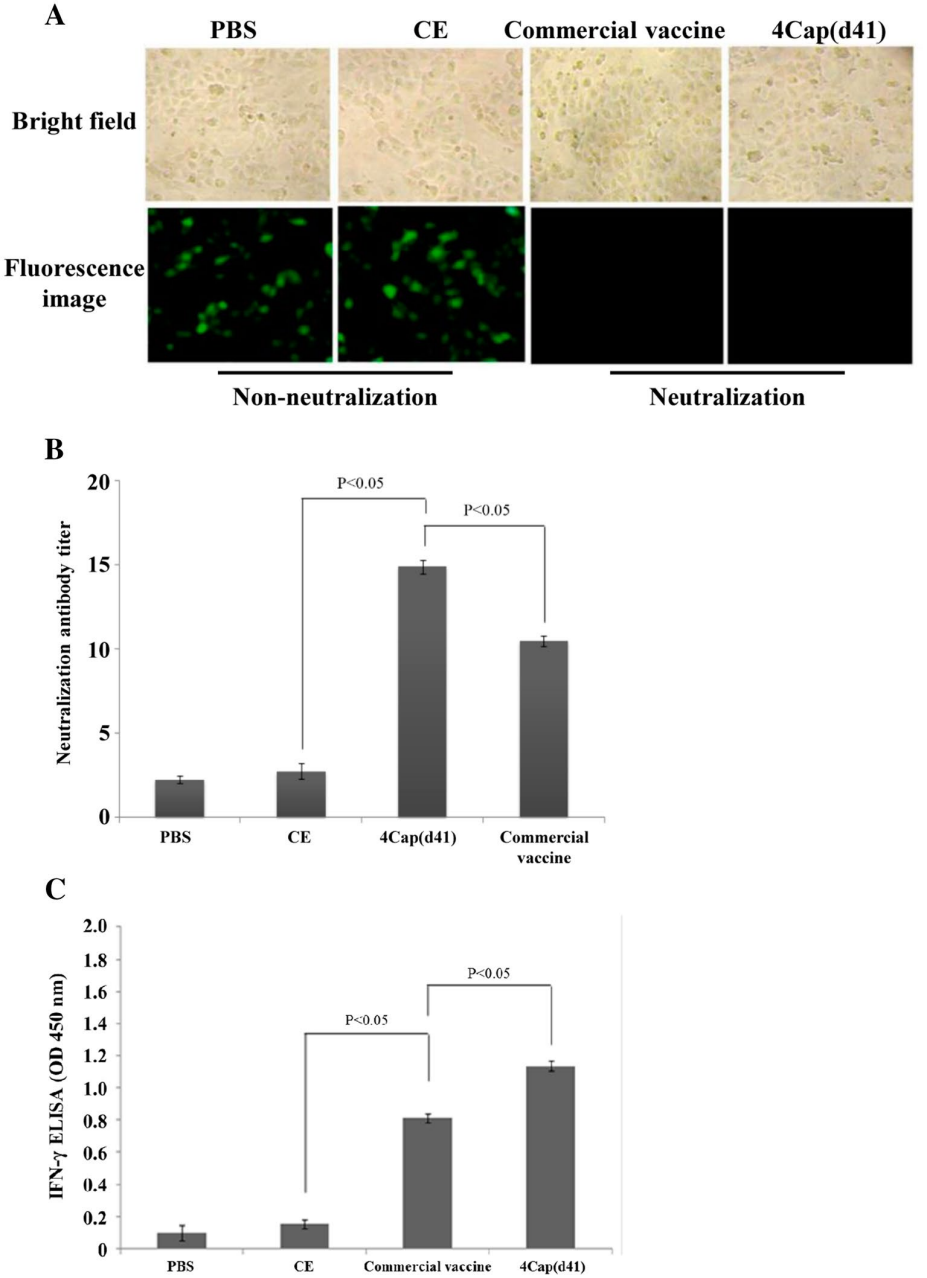
**Serum neutralization (SN) titers and the level of IFN-γ in mice immunized with various immunogens. A** The SN titers in mice induced by 4Cap(d41) and commercial vaccines. 5 × 10^7^ Sf-9 cells were infected with the recombinant baculovirus BacDD-4Cap(d41) at an MOI of 10 for 3 days. Cell lysates were collected to prepare the 4Cap(d41) vaccine. Eight-week-old mice were vaccinated via intraperitoneal injection at weeks 0 and 2 with PBS, CE, 4Cap(d41), and commercial vaccines as formulated with complete and incomplete adjuvants. As negative controls, three mice were injected with PBS and CE (5 × 10^7^ BacCE-infected Sf-9 cell lysates). Each mouse received one booster shot in week 2, and blood samples were taken at week 5 for the SN titer assay. **B** The fluorescence results were converted into neutralizing antibody titers and presented as a data chart (**B**). Data represent the mean ± SD. **C** Analysis of IFN-γ by ELISA was performed using the IFN-γ ELISA Kit. The value of OD_405_ nm was measured with an ELISA reader. Data represent the mean ± SD. A P value less than 0.05 was considered significant.

In this work, fluorescence staining was observed under a magnification of 200× under a fluorescent microscope (Figure 5A). The fluorescence results were converted into neutralizing antibody titers and presented as a data chart (Figure 5B). The results showed that neutralizing antibody titers induced by the commercial and 4Cap(d41) vaccines were 1:11 and 1:14, respectively. The virus neutralization titers of mice immunized with the commercial and 4Cap(d41) vaccines were much higher than those of the negative groups (CE and PBS). This result is consistent with the PCV2 neutralization titer 1:14 reported previously [29].

**Figure 6:**
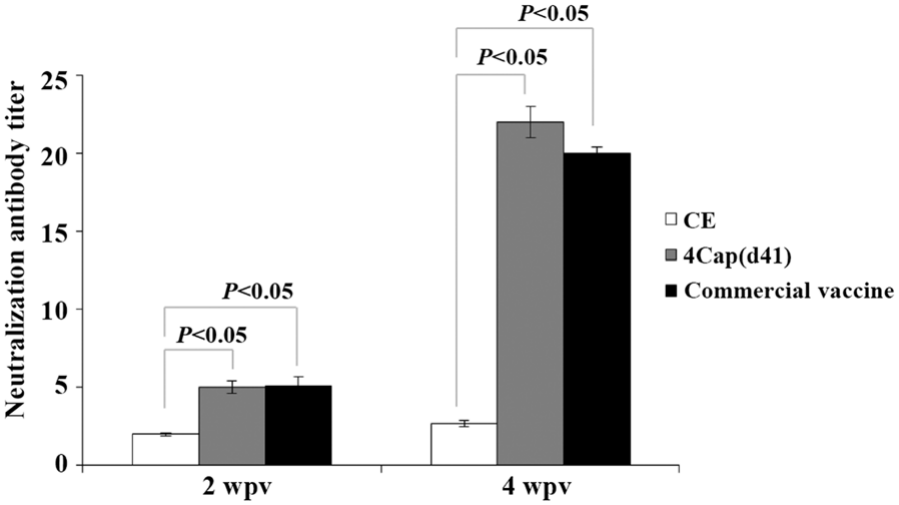
**Serum neutralization (SN) titers in swine immunized with various immunogens.** 2-month-old SPF pigs were immunized at the base of the ear by the intramuscular route with PBS, CE, 4Cap(d41) (80 μg), and commercial vaccines, respectively. Sera samples were collected at 14 and 28 days after primary immunization for virus neutralization tests. Data represent the mean ± SD. A P value less than 0.05 was considered significant

As shown in Figure 6, swine immunized with 4Cap(d41) and commercial vaccines triggered PCV2 neutralizing antibody titers of 1:5.5 and 1:5 at 2 weeks after primary immunization, which increased further to 1:22 and 1:21 at 4 weeks post vaccination.

### Change of the ratio of CD4^+^ to CD8^+^ in pig PBMC cells

To determine whether the 4Cap (d41) vaccine can cause T lymphocyte subsets in pigs, we collected PBMC cells from the blood of immunized pigs and analyzed them by flow cytometry. As shown in Table 2, the percentage of CD4^+^ T cells in the 4Cap(d41) group and the commercially available vaccine group was significantly higher than in the CE negative control group. The percentage of CD8^+^ T cells in 4Cap(d41) group and commercial vaccine group was significantly lower than in CE group. The ratio of CD4^+^/CD8^+^ in 4Cap(d41) and commercial vaccine groups was significantly higher than that in the CE negative control group.

**Table 2:** **Analysis of CD4**^**+**^ **and CD8**^**+**^ **of immunized pig PBMC cells by flow cytometry**

Group	CD4^+^ (%)	CD8^+^ (%)	CD4^+^/CD8^+^ ratio
CE	24.2 ± 0.49	5.9 ± 0.93*	4.14 ± 0.57
4Cap(d41)	39.5 ± 0.7*	1.5 ± 0.04	27.1 ± 1.16*
Commercial vaccine	21.9 ± 0.85	1.2 ± 0.04	18.74 ± 1.4*

The symbol (*) shows statastical differences between CE group and experimental groups (p < 0.05)

### Lymphocyte proliferative responses in swine immunized with different immunogens

**Figure 7:**
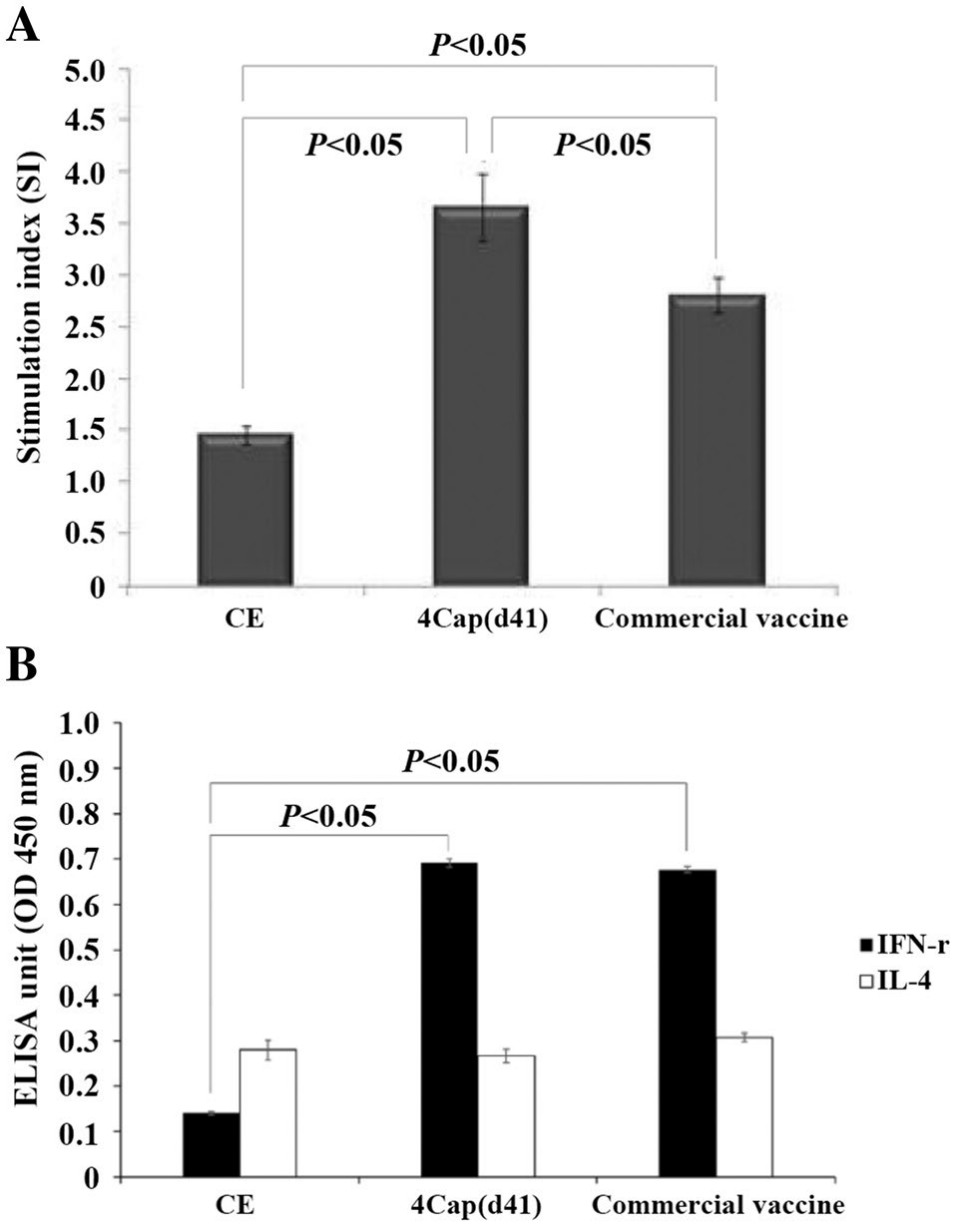
**Lymphocyte proliferation assay and analysis of IFN-γ and IL-4 by ELISA. A** Lymphocyte proliferation assay was done using PBMCs of swine as described in “Materials and methods” section. The optical density (OD) was determined at 490 nm, and the stimulation index (SI) was calculated as follows: SI = mean OD of PCV2 stimulated cells/mean OD of unstimulated cells. **B** Analysis of IFN-γ and IL-4 by ELISA was performed using the IFN-γ and IL-4 ELISA Kit. The value of OD_405_ nm was measured with an ELISA reader. Data represent the mean ± SD. A P value less than 0.05 was considered significant.

In the present study, we investigated whether the 4Cap (d41) vaccine can effectively induce cellular immune responses in pigs after two immunizations. To investigate whether the pseudotyped baculovirus BacDD-4Cap(d41) can induce cell-mediated immune responses, the lymphocyte proliferative responses were examined at 42 days after primary immunization. As shown in Figure 7A, the SI value (3.75 ± 0.3) was significantly higher in swine immunized with the 4Cap(d41) vaccine than those immunized with commercial vaccine (2.7 ± 0.15) and the negative control CE (1.4 ± 0.1). The difference was statistically significant (P < 0.05). These results suggested that the 4Cap(d41) vaccine prepared from genetic recombinant baculovirus BacDD-4Cap(d41)-infected Sf-9 cells induced a remarkable cellular immune response in swine.

### Analysis of IFN-γ in swine immunized with different immunogens

A previous study showed that IFN-γ is an important factor in the vaccine-induced cellular immune response [30]. Therefore, IFN-γ was used as an indicator of cellular immunity in this study. It was confirmed by IFN-γ ELISA that both 4Cap (d41) and commercially available vaccines can stimulate immune cells to secrete IFN-γ and induce cellular immune responses (Figures 5C, 7B). Although 4Cap (d41) and the commercially available vaccine group produced IL-4, there was no significant difference compared to the negative group CE (Figure 7B).

### Analysis of the serum antibody level of pigs immunized with 4Cap (d41) and commercial vaccines

**Figure 8:**
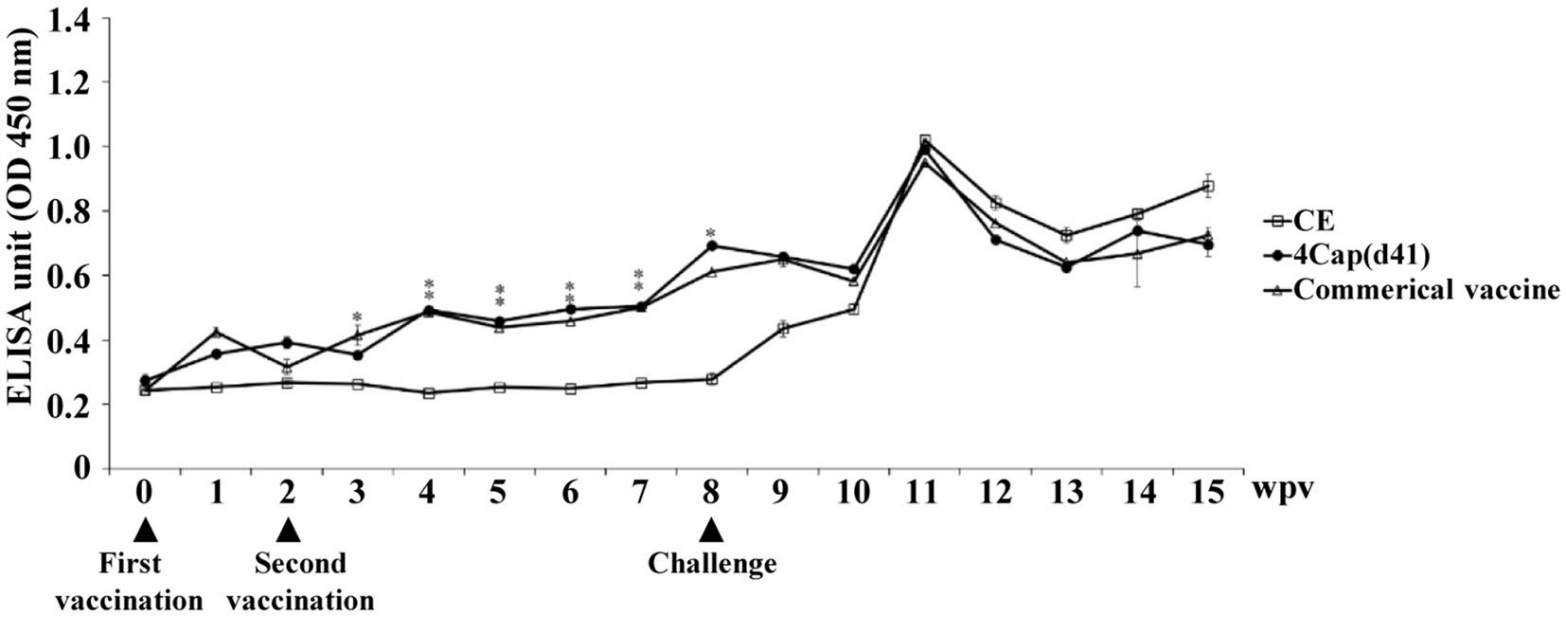
**Detection of anti-PCV2 titers by ELISA.** 2-month-old SPF pigs were immunized at the base of the ear by the intramuscular route with CE, 4Cap(d41), and commercial vaccines, respectively. Serum samples were collected every week after the first immunization to determine the Cap-specific ELISA antibodies. In the eighth week after the first immunization, all vaccinated pigs were intranasally injected with 2 ml of PCV2 (10^5.7^TCID_50_/mL). Data represent the mean ± SD. An asterisk (*) shows a statistically significant difference between the indicated groups (P < 0.05).

In the group vaccinated with 4Cap(d41) and commercial vaccine, the antibody levels in the eighth week after the first immunization gradually increased to reach the highest OD_405_ nm of 0.7 and 0.62, respectively, which was significantly different from the CE negative control group (Figure 8). The challenge test was conducted in the eighth week after the first immunization. The antibody levels of the 4Cap(d41) vaccine group, the commercial vaccine group, and the CE negative control group reached the highest OD_405_ nm of 1.0, 0.96 and 1.0 in the third week after challenge. These data indicate that the challenge in pigs will affect the increase in antibody levels.

### Quantitative analysis of PCV2 DNA in pig blood by real-time quantitative PCR

**Figure 9:**
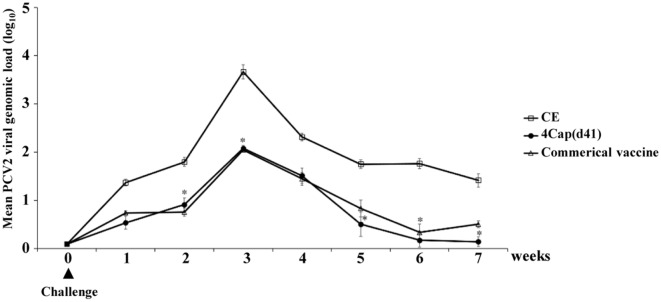
**Quantification of PCV2 viral loads in sera from the challenged pigs.** 2-month-old SPF pigs were immunized at the base of the ear by the intramuscular route with CE, 4Cap(d41), and commercial vaccines, respectively. Serum samples were collected every week after the first immunization. In the eighth week after the first immunization, all vaccinated pigs were challenged via intranasal injection with 2 ml of PCV2 (10^5.7^TCID50/mL). The 8^th^ week after the first immunization is designated as the 0^th^ week. PCV2 viral loads were detected by real-time quantitative PCR one week post challenge. Data are presented as mean ± SD. Viral loads were determined as the mean of the logarithmic DNA copy number per ml (log copies/ml). An asterisk (*) shows a statistically significant difference between the indicated groups (P < 0.05).

In the third week after the challenge, the PCV2 viral load reached the highest level. The viral load Log_10_ of the 4Cap(d41) vaccine group was 2.11; the viral load Log_10_ of the commercial vaccine group was 2.12 (Figure 9). In the seventh week after the challenge, the viral load Log_10_ of the 4Ca(d41) and commercial vaccine groups was 0.11 and 0.4, respectively while the viral load of the CE negative control group was is 1.57 (Figure 9).

## Discussion

PCV2 is a major infectious pathogen that causes PMWS, which is one of the most important swine diseases that causes significant economic losses worldwide. The PCV2 Cap protein is the major structural viral protein encoded by ORF2 and induces host-specific neutralizing antibody [4–6], which makes it an important target in the design of a subunit vaccine against PCV2 infections. Several studies have been conducted on PCV2 vaccines [31–37]. One previous study confirmed that the N-terminal 47 amino acid is not related to the structure of the epitope [38], and its N-terminal 41 amino acids have been confirmed to contain a nuclear localization signal (nuclear localization signal; NLS), where the N-terminal 12–18 amino acid (RHRPRSH) and 34–41 amino acid (HRYRWRRK) fragments are regarded as important signals for transport to the nucleus [39]. Another report pointed out that the PCV2 Cap protein with NLS will be transported to the nucleus after translation. When this NLS is deleted, the Cap protein is retained in the cytoplasm [23, 39]. To prevent the nuclear localization signals (NLS) of the Cap protein from affecting cell viability and Cap protein displayed on cell membranes by the baculovirus surface display system, the first 41 amino acids at the N-terminus of the PCV2 Cap protein were deleted and the PCV2 Cap(d41) protein was expressed/displayed on cell membranes. It was found that the Cap(d41) protein contains two fragments with different molecular weights. In low MOI, low levels of the Cap(d41)-gp64 (SP-TM-CTD) fusion protein was expressed and sent to the cell membrane, thus most of gp64 SP fragment has been excised from the Cap(d41)-gp64 (SP-TM-CTD) fusion protein. Thus only one major band (molecular weight 28 kDa) was seen. In the high MOI group, the higher levels of the Cap(d41)-gp64 (SP-TM-CTD) fusion protein were expressed. When the cell membrane with displayed protein has reached saturation, it may accumulate excess protein in the cytoplasm (molecular weight 33 kDa). Therefore, two bands were seen in the high MOI group.

The recombinant baculovirus surface display system has been exploited for the production of vaccines [20–22, 36, 40, 41]. However, the higher costs of using eukaryotic expression systems are the obstacles when the products are developed for commercial application. In the present study, we have successfully constructed three novel baculovirus surface display vectors with multiple expression cassettes for high level expression of the PCV Cap(d41) protein. In this work, coexpression of EGFP allowed rapid identification of the recombinant baculovirus in Sf-9 insect cells, eliminating cumbersome and time-consuming assays [42]. As the number of expression cassettes in the recombinant baculovirus increases, the expression levels of Cap(d41) protein also increased. The higher expression levels of the Cap(d41) protein with the recombinant baculovirus BacDD-4Cap(d41) is about twice that of BacSC-Cap(d41). It is curious that the expression levels of the PCV2 Cap (d41) protein may not necessarily be the same as the number of expression cassettes of the vector increase. We found that the expression level of the PCV2 Cap(d41) protein by recombinant virus BacDD-3Cap(d41) is close to the expression level of Cap(d41) protein by recombinant virus BacDD-4Cap \(d41). It is speculated that the reason may be that multiple expression cassettes are constructed using the p10 or polh promoters, which control expression of the p10 and polyhedrin proteins, which are expressed concurrently during the late stage of infection. This could result in competition between promoters leading to decreased expression efficiency. This hypothesis is further supported by an earlier study suggesting that expression from the p10 promoter inhibits the transcriptional activity of the polh promoter [43].

This study also tested the optimal conditions for expressing the Cap(d41) protein with the recombinant baculoviruses BacSC-Cap(d41), BacDD-2Cap(d41), BacDD-3Cap(d41) and BacDD-4Cap(d41). Our results reveal that these viruses yield low levels of protein expression on the second day after infection of the cells, and the highest levels on the fourth day after infection. Although Cap(d41) protein expression is the highest on the fourth day after virus infection, serious cytopathic effect (CPE) was observed, which may affect the quality of Cap(d41). The cells after infection must maintain their integrity to ensure the quality of the expressed protein. One previous study found that expression of exogenous proteins in insect cells causes the degradation of exogenous proteins. The rate of protein synthesis is the same as the rate of degradation 1 day after infection. The degradation does not disappear until 3–4 days after infection in insect cells [44]. This may explain why after 2 days of infection with insect cells, the PCV2 4Cap(d41) protein was still not detected (Figure 4A). Due to serious cytopathic effect (CPE) and the increase in production costs on the fourth day after virus infection, we conclude that 3 days of infection is more suitable for protein production. Among the MOI tested, both 5 and 10 MOIs have higher levels of protein expression. Furthermore, we also found that 10^7^ of Sf-9 cells infected with the recombinant baculovirus BacDD-4Cap (d41) produced higher expression levels of Cap(d41) protein and elicited higher levels of ELISA titers than that of cell number 10^5^ and 10^6^ of Sf-9 cells. Thus, this study suggests that incubating virus-infected cells for 3 days, 5–10 MOI, and cell number of 10^7^ Sf-9 cells are the optimal conditions for mass production of the Cap (d41) protein.

An effective vaccine must induce both humoral and cellular immune responses to prevent diseases. In the present study, a virus neutralization test confirmed that pigs immunized with the 4Cap(d41) vaccine can induce a neutralizing antibody titer of 1:22. We found that the 4Cap(d41) vaccine significantly reduces the viral load of vaccinated pigs (Figure 9). These data are consistent with a previous report suggesting that a neutralizing antibody titer of 1:11 can effectively inhibit viremia [40]. Although the PCV change induced similar levels of antibodies in CE, 4Cap and commercial vaccines, our results showed that the 4Cap(d41) and the commercial vaccine groups significantly reduce the viral load of vaccinated pigs as compared with the CE negative control group. This could be 4Cap(d41) and commercial vaccines-induced higher levels of neutralizing antibody in mice and SPF pigs than that of the CE negative control group (Figures 5, 6).

Previous studies have shown that IFN-γ is regarded as an important factor in the vaccine-induced cellular immune response [30]. Therefore, IFN-γ was used as an indicator of cellular immunity in this study. CD4^+^T lymphocytes can differentiate into Th1 and Th2. Th1 secretes IFN-γ to activate CD8^+^T lymphocytes and macrophages to kill foreign pathogens or infected cells. It was confirmed by IFN-γ ELISA that both 4Cap(d41) and commercially available vaccines can stimulate immune cells to secrete IFN-γ and induce cellular immune responses. This study reveals that the recombinant 4Cap(d41) group and the commercially available vaccine group can increase the number of CD4^+^ T cells and the ratio of CD4^+^/CD8^+^ and induce a significant higher levels of cellular immune response.

Our data reveal that both 4Cap(d41) and commercially available vaccines can induce the Th1 immune response as evidenced by higher levels of IFN-γ production. This study shows that cell lysates from the recombinant baculovirus BacDD-4Cap(d41)-infected cells can trigger better humoral and cellular immune responses than that of BacSC-Cap(d41). This study confirmed that the Cap(d41) protein with 41 amino acids deleted at the N-terminus can be stably expressed in SF-9 cells. We successfully constructed a recombinant baculovirus BacDD-4Cap(d41) carrying 4 expression cassettes to enhance protein expression. The optimal conditions for mass production of the Cap(d41) protein are established, including incubating virus-infected cells for 3 days, 5–10 MOI, and cell number of 10^7^ Sf-9 cells for virus replication. In animal experiments, the 4Cap(d41) vaccine prepared from BacDD-4Cap(d41)-infected cells can trigger better humoral and cellular immune responses.

## Data Availability

The datasets supporting the conclusions of this article are included within the article.
